# The views and practice of oncologists towards nutritional support in patients receiving chemotherapy

**DOI:** 10.1038/sj.bjc.6603280

**Published:** 2006-08-01

**Authors:** A Spiro, C Baldwin, A Patterson, J Thomas, H J N Andreyev

**Affiliations:** 1 Department of Medicine and Therapeutics, Imperial College Faculty of Medicine, Chelsea and Westminster Hospital, London, UK; 2 The Gastrointestinal Unit, Department of Medicine, Royal Marsden Hospital, Fulham Road, London SW3 6JJ, UK; 3 The Gastrointestinal Units, Royal Marsden Hospital, Surrey, UK; 4 Department of Nutrition and Dietetics, King's College London, London, UK

**Keywords:** weight loss, nutritional assessment, physician

## Abstract

Malnutrition in patients with cancer is common and an adverse prognostic indicator. A questionnaire answered by 357 (72%) UK specialist oncological trainees suggests that they lack the ability to identify factors that place patients at risk from malnutrition. Major barriers to effective nutritional practice included lack of guidelines, knowledge and time.

As long ago as 1932, malnutrition was identified as a prognostic indicator of the outcome in cancer patients (Warren, 1932). Up to 80% of patients with cancer are malnourished at presentation (Dewys *et al*, 1980; O?Gorman *et al*, 1998), and in up to 20%, malnutrition is a significant contributing factor to their death (Ottery, 1996). Studies show poorer response to treatment, a reduced quality of life and increased risk of death in those patients who have lost weight (Oveson *et al*, 1993; Andreyev *et al*, 1998; Ross *et al*, 2004).

Best practice, as stated by NICE Guidelines requires that patients should undergo nutritional assessment so that those shown to be at risk can be considered for treatment (National Institute for Clinical Excellence, 2006). The publication *Nutrition and patients: a doctor's responsibility* (Kopelman and Lennard-Jones, 2002) set out to raise awareness of the fundamental importance of nutritional care in everyday clinical practice. Yet, there is overwhelming evidence to suggest that few doctors deal with malnutrition adequately (McWhirter and Pennington, 1994; Edington *et al*, 2000; Kelly *et al*, 2000; Beck *et al*, 2002). An understanding of health professionals? attitudes to nutrition, particularly those of oncologists who look after patients with the highest prevalence of malnutrition, is important if it is to be recognised efficiently and steps taken to address it.

The aims of the study were three-fold: to develop an understanding of the extent to that oncologists are able to identify malnutrition, to elucidate the importance which oncologists place on nutrition as a variable in the clinical care and outcome of their patient and to identify the barriers that might exist in the decision to advocate nutritional support.

## MATERIALS AND METHODS

A case-scenario-based questionnaire was developed and piloted to address three issues: (1) the identification of malnutrition, (2) the importance of nutritional status and support and (3) the barriers preventing nutritional intervention. Two case scenarios in patients with gastrointestinal cancer were used, the first related to identification of malnutrition and the second to the role and indication for nutritional support for a patient who had lost weight. Additionally, their views on the importance of various factors in treatment outcome and confidence in assessing malnutrition were assessed.

The final version of the questionnaire was piloted in all specialist oncological trainees at one centre. Subsequently, on the basis of the responses recorded, it was decided to send it out to all UK trainees, identified by their membership of the Association of Cancer Physicians, UK or the Royal College of Radiologists, UK. The scenarios were content validated by a group of defined UK experts on malnutrition, who set the expert standard.

Results were analysed using SPSS v13. Frequencies were described and *χ*^2^ tests were used to assess whether there were associations between nutritional practice, knowledge and attitudes and clinical speciality, nutritional education or years of clinical and oncology experience. Significance was established at *P*<0.05.

## RESULTS

Between April and June 2003, 61 pilot questionnaires were distributed to trainees in one institution. Subsequently, between September 2004 and April 2005, a further 433 questionnaires were sent out to all trainees in the UK. Of 494 questionnaires in total, 357 were returned (72% response rate). Of these, six were not completed because the recipient was no longer working in oncology, and 14 because they were not available at the given address. Of the 337 completed, the maximum missing data for any scenario response on completed questionnaires was less than 2% (*n*<7). Nineteen questionnaires were sent out to experts, of whom 16 replied (84%). The characteristics of the responders are shown in Table 1.

### Do oncologists consider nutrition important to outcome?

Almost all specialist oncological trainees thought that ?stage? or ?performance status? was very important to the outcome, but nearly two-thirds (65%, *n*=217) rated nutritional status as very important. Age and patient attitude were rated as much less important (Table 2a).

In the case study scenario, nearly all trainees thought that the patient's morbidity and quality of life would be affected by nutritional intervention. A substantial majority also felt that nutrition intervention would play a role in hospital stay (76%, *n*=255) and treatment toxicity (78%, *n*=261), but a larger number indicated uncertainty. Trainees were least likely to agree that nutritional intervention would play a role in mortality with regard to this patient (Table 2b).

### Can oncologists identify malnutrition?

The majority of specialist oncological trainees (80%, *n*=267) expressed uncertainty or a lack of confidence in their ability to identify malnutrition. Those who had undergone undergraduate nutritional lectures were more confident (*P*<0.01), but no association was found between confidence and speciality (medical *vs* clinical oncologist) age, medical or oncological experience or type of hospital was seen.

There was a discrepancy (Table 3a) between trainees who significantly more frequently identified the case patient as definitely malnourished in comparison to experts (*P*<0.05).

When asked which variables they would find useful to assess nutritional status (Table 3b), 48% (*n*=160) of trainees failed to specify height and/or body mass index. Just over one-quarter of trainees identified the additional variables necessary to identify risk according to the Malnutrition Advisory Guidelines (MAG), Malnutrition Universal Screening Tool (?MUST?) criteria or the Malnutrition Screening Tool (MST), compared to over three-quarters of experts. A similar pattern was shown by trainees (29%, *n*=97), in recording half or more of the six variables required to identify nutritional risk according to the Patient Generated Subjective Global Assessment (PG-SGA), a specific and validated tool for assessing cancer patients? nutritional status. The ability of oncologist trainees to identify relevant variables was associated with undergraduate nutrition lectures (*P*<0.05) but not with medical or oncological experience.

When asked to identify the level of weight loss in a 1-month period, which indicated that nutritional intervention was necessary (Table 3c; case scenario 2), again specialist oncological trainees gave significantly different replies to experts (*P*<0.05), who considered nutritional intervention as necessary at a lower level of weight loss than the trainees.

### What barriers prevent inclusion of nutrition in oncologist patient care?

As shown in Table 4, the three principal barriers to nutritional intervention by specialist oncological trainees were reported to be lack of clear guidelines (*n*=231, 69%), lack of knowledge (*n*=201, 60%) and lack of time (*n*=188, 56%). Two hundred and seventy (80%) oncological trainees wanted additional training in this area.

## DISCUSSION

The study suggests that oncologist trainees accept that nutritional status and nutritional intervention are important to outcome in patients receiving active therapy for malignancy. However, there is an inability to identify patients at risk of malnutrition and to refer those who may benefit from early nutritional intervention. Further barriers include a lack of recognised guidelines as to when to recommend nutritional intervention for weight loss.

Timely and appropriate interventions for patients with cancer require adoption of routine nutritional screening and evaluation (Ottery, 1995). Yet, hospital surveys suggest nutritional risk screening and assessment as part of routine practice is generally not performed (Duncan and Silk, 1997; Kondrup *et al*, 2002). It has been shown that malnutrition is largely unrecognised by health professionals (Edington *et al*, 2000). Similar findings more recently have come from The Council of Europe Group survey on nutritional care in European hospitals (Beck *et al*, 2002). Our study suggests that these findings on generalised hospital populations are also relevant in the oncological setting. This is particularly important as oncological treatment is increasingly given in the ?outpatient? setting where any standard ward-based nutrition assessment tool is not typically used. This study suggests that oncology trainees fail to identify patients appropriately for nutritional assessment, not because they think it is unimportant but rather because of lack of ability, confidence and knowledge of important criteria, which should determine effective nutritional practice.

There are limitations inherent in the questionnaire as a method of survey. Ideally, stringent methods of validation and reliability testing are required. However, our questionnaire was developed after a pilot study. This study is also limited in that it addresses the outcome at which behaviour is directed rather than the actual behaviour. Further research would need to ascertain actual rather than reported nutrition practice.

The study suggests that future research also needs to be directed at the best method of providing effective, concise and relevant nutritional education interventions to oncologist trainees.

In conclusion, oncologists lack the ability to identify factors that place patients at risk from malnutrition. Although oncologists acknowledge the importance of nutritional support, barriers such as lack of knowledge, clear guidelines and lower priority because of time constraints may prevent referral for, or direct nutritional intervention. Until the ethos of optimal nutritional management is strengthened in clinical practice, probably through continuing effective education and training at all levels within the medical profession, the rate of untreated malnutrition may remain unacceptably high and continue to compromise patient outcomes.

## Figures and Tables

**Table 1:** Respondent characteristics

	**Oncologist SpR**, ***n* (%)**
*Gender*	
Male	144 (37)
Female	210 (62)
Not indicated	3 (<1)
	
*Age (years)*	
⩽30	62 (18)
31?34	175 (52)
35?39	77 (23)
⩾40	17 (5)
Not indicated	6 (2)
	
*Specialisation*	
Medical Oncologist	139 (41)
Clinical Oncologist	182 (54)
Surgery	4 (1)
Palliative Care	2 (<1)
Pediatrics	3 (<1)
Hematology	1 (<1)
GP	1 (<1)
Not indicated	5 (2)
	
*Hospital*	
District General	25 (7)
Teaching	143 (42)
Tertiary	163 (48)
Not indicated	6 (2)
	
*Place of training*	
UK	286 (85)
Europe	24 (7)
Australia	4 (1)
South Asia	11 (3)
South Africa	4 (1)
Middle East	2 (<1)
West Indies	1 (<1)
Not indicated	4 (1)
	
*Clinical experience (years since full medical registration)*	
<10	194 (58)
⩾10	138 (41)
Not indicated	5 (2)
	
*Oncologic experience (years working in oncology)*	
<5	174 (52)
⩾5	158 (47)
Not indicated	5 (2)
	
*Nutritional education*	
Undergraduate lectures	118 (35)
Postgraduate education	35 (10)
	
Interest in further nutritional training	270 (80)

**Table 2:** Do trainee oncologists consider nutrition important to outcome?

**(a) Importance of different factors to outcome (0 not important?5 very important)** (**total completed 334**)			
	**4?5 response, *n* (%)**	**Median**	**Range**
Stage	321 (96)	5	3?5
Performance status	324 (97)	5	3?5
Nutritional status	217 (65)	4	1?5
Age	124 (37)	3	0?5
Patient attitude	127 (38)	3	0?5
			

**(b) Importance of nutritional intervention to outcome ?In a patient with 11% weight loss would nutritional intervention play a role? *(Yes, No, Uncertain)*** (**total completed 335)**			
	**Yes, *n* (%)**	**No, *n* (%)**	**Uncertain, *n* (%)**
Mortality	188 (56)	57 (17)	90 (27)
Morbidity	305 (91)	7 (2)	23 (7)
Hospital stay	255 (76)	10 (3)	67 (20)
Quality of life	318 (95)	0 (0)	17 (5)
Toxicity from treatment	261 (78)	27 (8)	47 (14)

**Table 3:** Can trainee oncologists identify patients at risk of malnutrition?

**Case Scenario 1:**		
**69-year-old female with GI tumour, third cycle chemotherapy, weight 54 kg, albumin 25 g l^−1^, CRP 18 mg l^−1^, other biochemistry normal**		
	**Oncologist, *n* (%)**	**Expert, *n* (%)**
*(a) Is this patient:*		
Definitely malnourished	137 (41)	2 (14)
At risk of malnutrition/cannot be assessed from the information given	197 (59)	14 (86)
		
*(b) What further variables would be required for nutritional assessment?*		
Height and/or BMI	174 (52)	15 (94)
BMI and weight history (MAG tool)	97 (29)	13 (81)
		

**Case Scenario 2:**		
**56-year-old male with GI tumour to commence chemotherapy, biochemistry normal, no comorbidity weight 62 kg, usual weight 70 kg**,		
*(c) What weight loss over 1 month indicates need for nutritional intervention*		
1?4% (or kg eq)	10 (3)	1 (7)
5?9% (or kg eq)	150 (45)	12 (75)
10?14% (or kg eq)	157 (47)	3 (21)
15+% (or kg eq)	10 (3)	0 (0)
No intervention	7 (2)	0 (0)

BMI=body mass index, CRP=C-reactive protein, eq=equivalent, GI=gastrointestinal, MAG=Malnutrition Advisory Guidelines.

**Table 4:**
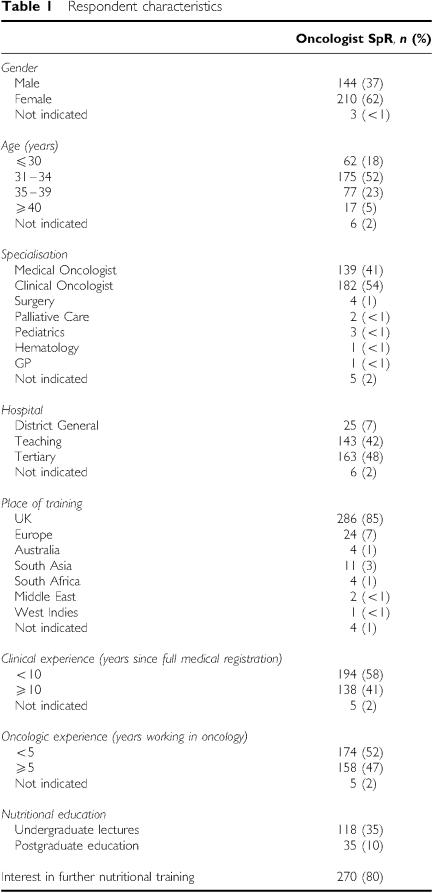
What barriers prevent inclusion of nutrition on oncologist patient care?
